# Burosumab treatment of a child with McCune–Albright syndrome/polyostotic fibrous dysplasia: challenges and benefits

**DOI:** 10.1093/jbmrpl/ziaf042

**Published:** 2025-03-10

**Authors:** Sophia D Sakka, Danai Georgakopoulou, Artemis Doulgeraki, Andreas H Krieg, John Anastasopoulos, Gabor Szinnai, Christina Kanaka-Gantenbein

**Affiliations:** Division of Endocrinology, Metabolism and Diabetes and Aghia Sophia Children’s Hospital Endo-ERN Center for Rare Pediatric Endocrine Disorders, First Department of Pediatrics, Medical School, National and Kapodistrian University of Athens, “Aghia Sophia” Children’s Hospital, Athens 11527, Greece; Division of Endocrinology, Metabolism and Diabetes and Aghia Sophia Children’s Hospital Endo-ERN Center for Rare Pediatric Endocrine Disorders, First Department of Pediatrics, Medical School, National and Kapodistrian University of Athens, “Aghia Sophia” Children’s Hospital, Athens 11527, Greece; Department of Bone and Mineral Metabolism, Institute of Child Health, Athens 11527, Greece; Department of Hip, Deformity, Tumor and Infection, Bone- and Soft-Tissue Tumor Center University of Basel (KWUB), Basel 4031, Switzerland; Second Department of Orthopaedics, “Aghia Sophia” Children's Hospital, Athens 11527, Greece; Department of Pediatric Endocrinology and Diabetology, University Children’s Hospital Basel, University of Basel, Basel 4031, Switzerland; Department of Clinical Research, University Hospital Basel, University of Basel, Basel 4031, Switzerland; Division of Endocrinology, Metabolism and Diabetes and Aghia Sophia Children’s Hospital Endo-ERN Center for Rare Pediatric Endocrine Disorders, First Department of Pediatrics, Medical School, National and Kapodistrian University of Athens, “Aghia Sophia” Children’s Hospital, Athens 11527, Greece

**Keywords:** fibrous dysplasia, McCune–Albright syndrome, burosumab, FGF23-mediated hypophosphatemia

## Abstract

Fibrous dysplasia/McCune–Albright syndrome (FD/MAS) is a rare condition caused by a mutation in the GNAS locus. Apart from endocrinopathies, some cases are characterized by excessive fibroblast growth factor 23 (FGF23) production from abnormal fibro-osseous tissue in FD lesions, resulting in increased renal phosphate excretion. We present a girl with FD/MAS and severe skeletal burden, evidenced by the presence of polyostotic fibrous dysplasia, which was complicated with bone fractures. She also had hyperthyroidism and GnRH-independent precocious puberty. She received zoledronic acid infusions in preparation for hip surgery. Despite optimal conventional management with oral phosphate and alphacalcidol, which was poorly tolerated, she presented persistent hypophosphatemia. To control hypophosphatemia and its deleterious effects on bone health, treatment with burosumab off-label at a dose of 0.66 mg/kg (20 mg) every 2 wk was initiated. Serum phosphate levels normalized within 2 wk of treatment. Laboratory results showed improvement in serum alkaline phosphatase (ALP) and PTH levels. After the second injection of burosumab, phosphate and PTH rose above the normal range with normal vitamin D levels; therefore, the interval between doses was increased to 3 wk, and calcium 500 mg daily was added. However, phosphate levels dropped again below normal range, so she had to return to 2-weekly injections of 20 mg. After 11 mo on burosumab, she remains with high normal phosphate levels and normal PTH and ALP values. Burosumab is well tolerated, with no adverse events to date. Burosumab is a human monoclonal antibody against FGF23 that reduces the risk of developing FGF23-mediated hypophosphatemia and its associated complications. Burosumab should be considered as an effective and safe alternative strategy for FGF23-mediated hypophosphatemia in FD/MAS for those who either cannot tolerate or do not respond to conventional therapy. To our knowledge, this is the fourth published case worldwide describing successful treatment with burosumab in FD/MAS.

## Graphical Abstract

**Graphical Abstract:**
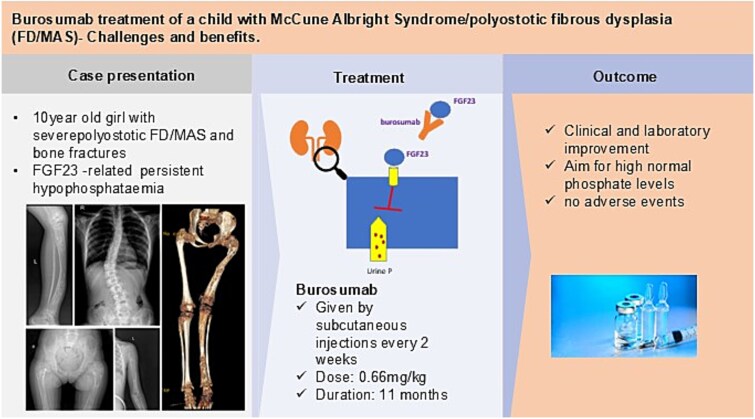

## Introduction

Fibrous dysplasia/McCune–Albright syndrome (FD/MAS) is a rare disorder of striking complexity, resulting from recurrent somatic gain-of-function mutations in the GNAS locus, which is located on chromosome 20q13.3. Activating Gsα mutations are associated with constitutive cAMP production, which leads to the autonomous endocrine hyperfunction observed in this syndrome.^1^

FD/MAS can involve a broad range of systems, resulting in a diversity of phenotypes.^2^ The combination of 2 or more classic features [FD of bone, café-au-lait skin macules, and/or associated hyperfunctioning endocrinopathies: gonadotropin-independent precocious puberty, non-autoimmune hyperthyroidism, growth hormone (GH) excess, hypercortisolism] is necessary to make the diagnosis.^2^^,^^3^

Frank hypophosphatemia in patients with FD is infrequent and is caused by overproduction of fibroblast growth factor 23 (FGF23) by abnormal osteogenic precursors in FD lesions. Elevated FGF23 reduces renal phosphate reabsorption, downregulates renal 1α-hydroxylase, which in turn decreases production of active 1,25-dihydroxyvitamin D [1,25(OH)2VitD] and increases PTH. Elevated PTH exacerbates the phosphate-wasting effect of FGF23 and deteriorates the disease.^4^

Treatment of frank hypophosphatemia has been traditionally similar to other disorders of FGF23 excess and includes oral phosphate supplements and calcitriol or alphacalcidol. Key treatment goals include healing of rickets, improving growth, relieving bone pain, and correcting leg deformities.^5^ However, oral phosphate leads only to a transient increase in serum phosphate levels. In turn, an increase in serum phosphate leads to a reduction of serum ionized calcium, causing secondary hyperparathyroidism. Long-term phosphate supplementation is associated with chronic stimulation of parathyroid hormone secretion, potentially leading to tertiary hyperparathyroidism. Active vitamin D analogs (calcitriol or alphacalcidol) can prevent hyperparathyroidism but increase the risk of hypercalciuria and nephrocalcinosis.^6^ Furthermore, phosphate supplements cause gastrointestinal discomfort and should be given four to five times daily, at long time distances from dairy products, which can interfere with the daily activities of the patients, impair compliance, and affect their quality of life.^3^ Moreover, conventional treatment increases FGF23 levels, potentially aggravating hypophosphatemia and 1,25(OH)2VitD deficiency.

Thus, treating these patients with burosumab, a fully human recombinant monoclonal antibody targeted to inhibit excess FGF23 bioactivity seems a very reasonable approach.^7^ Burosumab binds to and inhibits the activity of FGF23. By directly inhibiting excess FGF23, burosumab increases tubular reabsorption of phosphate from the kidney and, through the production of 1,25(OH)2VitD, enhances intestinal absorption of calcium and phosphate. Burosumab is indicated and recommended for the treatment of X-linked hypophosphatemic rickets (XLH), another disorder of FGF23 excess in children with radiographic evidence of rickets from 1 yr of age and in symptomatic adults.^6^

We present a 10-yr-old female patient with MAS and severe polyostotic FD who is being treated with burosumab.

## Materials and methods

**Figure 1:**
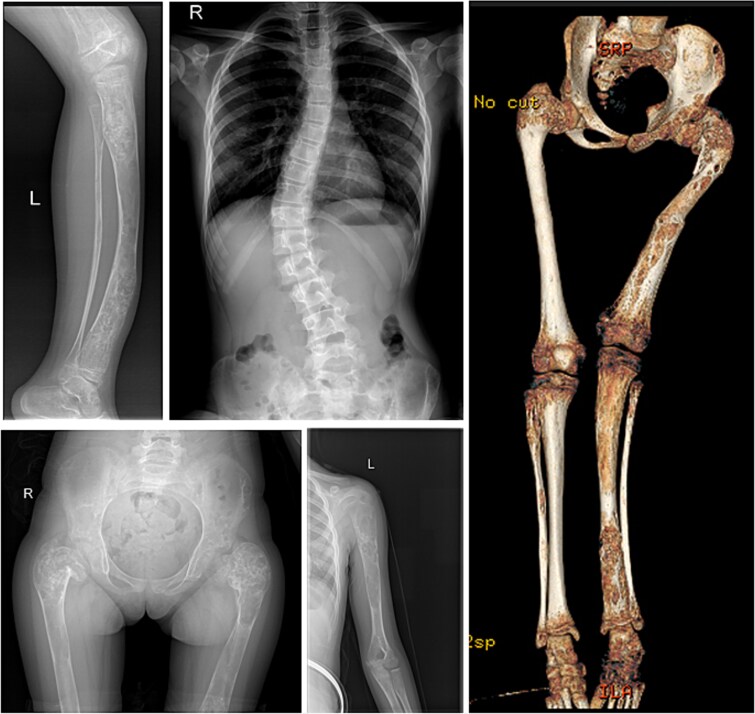
X-rays and 3D CT image of the patient showing the severity of the disease.

Our patient was first diagnosed with MAS at the age of 28 mo due to peripheral, GnRH-independent precocious puberty with premature menarche, bilateral cafe au lait lesions on her back, and FD lesions of both femurs, revealed on X-rays. Early in life she developed severe polyostotic FD with hypophosphatemia affecting most of her long bones (Figure 1) and causing various skeletal complications such as bone fractures, craniofacial deformity, and scoliosis. At the age of 17 mo, she suffered the first fracture of the left tibia, which was followed by a fracture of the left femoral head at the ages of 2.5 and 3 yr, a fracture of the left femoral neck at the age of 3.5 yr, and finally a fracture of the diaphysis of the left femur at the age of 8 yr. Taking into account her low BMD (Z-score aBMD L1-L4 = −0.5, Z-score total body less head aBMD = −2.4), the evidence of increased bone resorption (bTRAP 5b: 16.8 ng/mL, ref. range for age: 5.2-10.8 ng/mL, urine DPD/urine Cr: 119.7 mmol/mol, ref. range for age: 16-37 mmol/mol), and the need for multiple corrective surgical approaches, the decision has been set for her to receive 3 infusions of Zoledronic acid 0.025 mg/kg (in 6 mo intervals) before surgery. As a next step, she underwent orthopedic surgery on both legs (rodding of the left femur, left tibia, and right femur) in order to stabilize them and prevent further fractures.

Due to her GnRH-independent precocious puberty, she is also under treatment with aromatase inhibitors since the age of 2.5 yr. However, her bone age is advanced by 2 yr. Furthermore, she developed hyperthyroidism at the age of 3 yr and has been under treatment with carbimazole since then.

## Results

### Clinical management of hypophosphatemia

The girl exhibited frank hypophosphatemia with excess renal phosphate loss and raised ALP. Her ALP levels had started rising from the age of 3 yr, however, her phosphate levels only dropped below normal at the age of 6.5 yr when she was started on phosphate supplements (initial dose 30 mg/kg/d) and alphacalcidol (initial dose 25 ng/kg/d).

Subsequent increases in the doses of oral phosphate (max dose 100 mg/kg/d) and alphacalcidol (max dose 80 ng/kg/d) failed to control serum phosphate and ALP. The treatment could not be tolerated due to gastrointestinal disturbances and hypercalciuria. As expected, her phosphate requirements were particularly high around Zoledronate infusions. Also, as expected, she had severely elevated FGF23 levels [471kRU/L (ref. range 26-110)]. Therefore, in order to moderate the deleterious effects of uncontrolled hypophosphatemia on bone health, it was decided to treat her with burosumab off-label at a dose of 0.66 mg/kg (20 mg) every 2 wk after 1 wk off conventional treatment.

The burosumab dose chosen was based on the recommended dose for children with XLH of 0.8 mg/kg rounded up to the nearest 10 mg, which resulted in a starting dose of 20 mg (0.66 mg/kg) (Burosumab SPC).

Serum phosphate levels normalized within 2 wk of treatment (Table 1). Laboratory results showed improvement in serum ALP and PTH levels. After the second injection of burosumab, phosphate and PTH rose above the normal range while vitamin D levels remained in the normal range. Although not a common practice, we increased the interval between doses to 3 wk to decrease phosphate levels and the risk for ectopic mineralization. Moreover, to ensure adequate calcium intake, we added 500 mg of calcium daily to lower PTH levels. However, phosphate levels dropped again below the normal range, so she had to return to 2 weekly injections of 20 mg. After the eighth injection, cholecalciferol was added to the treatment schedule due to low 25(OH)Vitamin D levels. After the 13th injection of burosumab the interval between doses was increased to 2.5 wk to lower PTH levels and decrease the risk for ectopic mineralization. This resulted in a drop of PTH levels; however, phosphate levels started dropping as well, so we returned to 2 weekly intervals. After 11 mo on burosumab treatment, she has been stable on 2 weekly injections, and her ALP and PTH levels have normalized. Furthermore, she has no renal phosphate wasting, and her phosphate levels remain in the high normal range. There were no short-term adverse events associated with burosumab. There was no significant change in her height SDS.

**Table 1:** Laboratory values and medication doses during burosumab treatment.

	1st injection	2nd injection	3rd injection	4th injection	5th injection	8th injection	10th injection	12th injection	13th injection	15th injection	17th injection	9 months	11 months
**ALP (U/L) (ref. range 60-240)**	**736**	**614**	**487**	**496**	**442**	**425**	**415**	**413**	**408**	**407**	**402**	**398**	**348**
**Ca (mg/dL)** **(ref. range 8.2-11)**	10.2	10.1	9.9	10.1	9.9	10.2	10.2		10	10	10.1	10.1	9.6
**P (mg/dL)** **(ref. range 4-6)**	**2.4**	5.2	5.2	4.7	3.6	4.8	4.7	4.6	3.9	4.7	**3.8**	4.5	5.2
**Cr (mg/dL) (ref. range 0.2-1)**	0.37	0.31	0.27	0.31	0.33	0.3	0.32	0.36	0.32	0.39	0.45	0.42	0.41
**PTH (pg/mL) (ref. range 12-62)**	**63.5**	**99.3**	**78.2**	**97.6**	21	**75.8**	**66.6**	**64.1**	55	48 (ref. range 22-88)		**72.7**	65.9 (ref. range 22-88)
**25(OH)VitD (ng/mL)**	25.6				44.2	**19**		25.4	23.26			41.7	32
**1,25(OH)D (pg/mL)** **(ref. range 18-65)**	27.3							26.2				36.2	
**FGF23 kRU/L** **(ref. range 26-110)**	**471**												
**Ca/Cr urine** **(ref. range 0.014-0.24)**	0.13	0.1	0.07	0.04		0.11	0.14	0.19	0.7		0.12	0.06	0.13
**TmP/GFR** **(ref. range 3.00–5.08)**	**2.39**		5.2	4.7		4.79	4.7		3.9	4.69	**3.03**	4.05	4.69
**Instructions/changes**	every 2 weeks	every 2 weeks	every 2 weeks	every 3 weeks	every 2 weeks	every 2 weeks	every 2 weeks	every 2.5 weeks	every 2.5 weeks	every 2.5 weeks	every 2 weeks	every 2 weeks	every 2 weeks
			calcium carbonate 500 mg/day			VitD 1200 IU/day			stop calcium				

Abnormal values are marked in bold.

## Discussion

To our knowledge, this is the fourth reported case of burosumab treatment in a child with FD/MAS in the international literature^8–10^ with a long follow-up period of 11 mo. According to the consensus statement from the FD/MAS international consortium in 2019, in all subjects with suspected polyostotic disease, baseline overnight fasting phosphate levels should be checked, and the calculation of the tubular reabsorption of phosphate (TmP/GFR) should be performed.^3^ The first step in the pharmacological management of FD is to ensure supplementation of phosphate if hypophosphatemia occurs, as well as vitamin D repletion. In our case, despite raised ALP levels and severe polyostotic FD, her phosphate levels only dropped below normal range at the age of 6.5 yr; therefore, conventional treatment was then initiated.

Hypophosphatemia due to FGF23 hypersecretion is generally more severe than in hyperparathyroidism due to lower 1,25(OH)2VitD levels. High serum FGF23, low serum 1,25(OH)2VitD, and adequate serum 25(OH)VitD should raise the suspicion of FGF23-mediated hypophosphatemia. Although vitamin D deficiency and FGF23-mediated hypophosphatemic disorders sometimes coexist, serum FGF23 levels are elevated in the latter condition.^7^

Because FGF23 is produced by dysplastic FD tissue, patients with higher skeletal FD burden, like our patient, have higher serum FGF23 than patients with low FD tissue involvement and subsequently lower phosphate levels.^11^ Moreover, dysplastic bone may be more vulnerable to the effects of hypophosphatemia than the normal bone in XLH.^12^ Therefore, burosumab, which is a targeted treatment for FGF23 excess, was given as an off-label treatment to our patient.

Low serum phosphate reduces hydroxyapatite formation causing osteomalacia, metaphyseal abnormalities, and impaired mineralization of growth plates causing rickets. These conditions lead to soft and often deformed bones, with skeletal abnormalities and impaired growth giving rise to lifelong pain and disability.^6^ In our case, almost every long bone was affected by FD, leading to multiple fractures that eventually needed to be rodded, so there was an imperative need to correct phosphate and reduce any risk factors affecting her bone health.

FD bone is inherently dysplastic and poorly mineralized, even in the absence of hypophosphatemia, which is a huge difference from XLH, where bone is normally formed. Gun et al. showed that patients with frank and low-normophosphatemia had higher intact FGF23 and ALP levels, higher Skeletal Burden Scores, increased long bone fractures and orthopedic surgeries, and a higher prevalence of moderate to severe scoliosis in comparison with those with high-normophosphatemia.^11^ It is therefore conceivable that targeting high-normal phosphorus levels may be the appropriate approach for patients with extensive FD lesions, which—in most cases—cannot be achieved with the association of vitamin D analog and phosphate supplementation.^11^ In our case, any attempt to increase her oral phosphate supplements in order to normalize her serum phosphorus levels, resulted in gastrointestinal complications affecting her compliance. However, burosumab monotherapy resulted in normal phosphorus levels for the first time and serum ALP that had never been so close to normal before. International consensus guidelines for XLH recommend titrating burosumab to achieve low-normal phosphorus levels (6). Of note, burosumab at a dose of approximately 0.8 mg per kilogram administered every 2 wk is an appropriate regimen for improving renal tubular phosphate reabsorption and clinical outcomes in children with X-linked hypophosphatemia.^13^ Current literature on burosumab is primarily focused on the more common genetic disorder X-linked hypophosphatemia (XLH). We chose to administer the recommended burosumab dose for XLH (0.8 mg/kg) and not for TIO (0.4 mg/kg), as there is larger evidence and experience on the use of this dose in children with XLH, compared to TIO, which is a condition that is rarely seen in childhood. As discussed above, there are unique considerations in the FD/MAS population that may necessitate a disease-specific approach. We started with the recommended SPC dose of 0.8 mg/kg every 2 wk that, from the beginning, resulted to high normal phosphate levels and normalized TmP/GFR. When we targeted high-normal phosphorus levels, PTH increased despite the addition of calcium and vitamin D supplementation, which was unfavorable. In case of increased PTH levels, 25(OH) vitamin D levels should be assessed and targeted between 30 and 50 ng/mL, and adequate nutritional calcium intake should be ensured. Preliminary studies have shown that prolonged anti-FGF23 treatment may stimulate PTH secretion, thus leading to the development of hyperparathyroidism. Persistent hyperparathyroidism despite vitamin D repletion and normal calcium nutritional intake should raise suspicion for treatment-related PTH hypersecretion or tertiary hyperparathyroidism associated with previous long-term phosphate supplementation.^5^ In our case, PTH normalized when intervals were spaced out and phosphate levels dropped, ruling out hyperparathyroidism. The levels of 1,25 vitamin D remained stable after 9 wk of treatment, and there was a mild increase after 12 wk. However, previous studies have shown a surge in 1,25 vitamin D levels between 3 and 7 d after the injection, which lessens with repeated doses to more physiologic levels.^14^^,^^15^ Therefore, it is possible that the specific time points chosen for 1,25(OH)2VitD assessment did not capture the expected surge.

Administering burosumab every 2.5 or 3 wk resulted in low-normophosphatemia, which also was undesirable. In our case, elevated PTH levels could be attributed either to previous long-term phosphate supplementation or to elevated phosphate levels targeted with burosumab. After returning to 2 weekly injections and continuing supplementation with calcium and vitamin D in order to maintain normal vitamin D and calcium levels, PTH and ALP finally normalized and phosphate remained at high normal levels.

**Figure 2:**
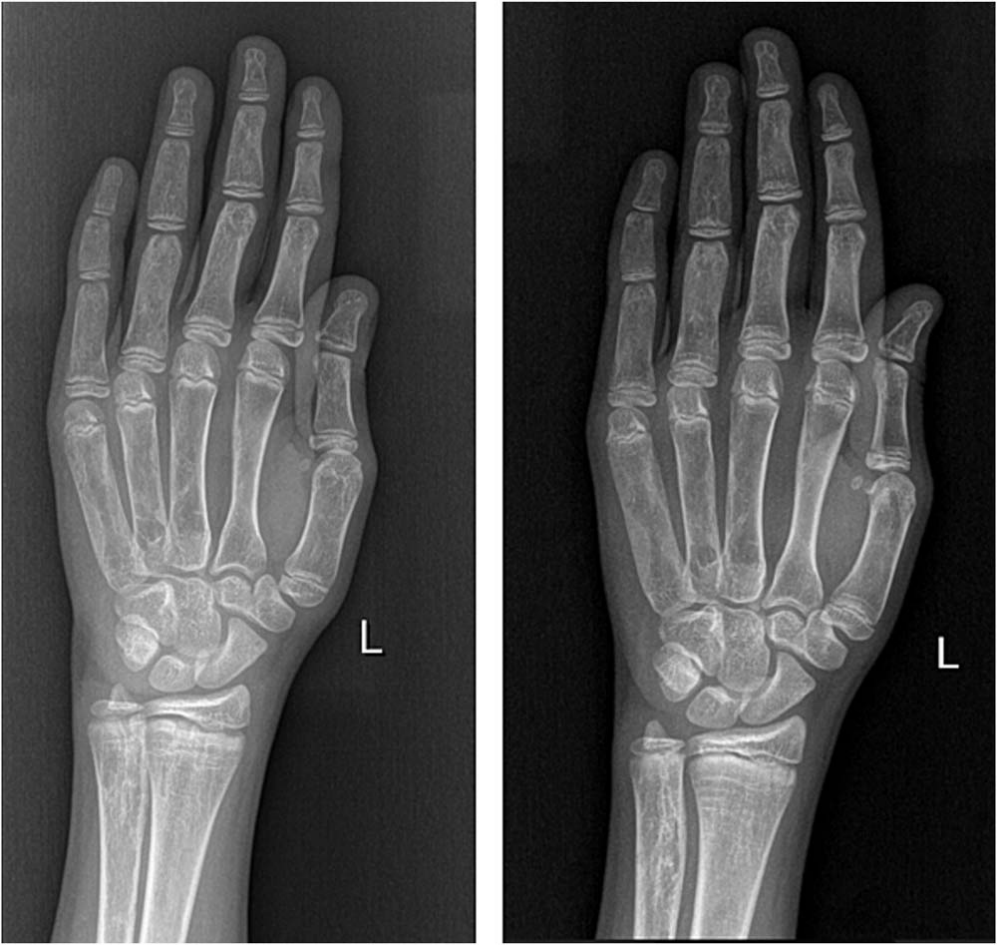
Hand X-rays 8 mo before (left) and 8 mo after starting of burosumab (right) showing mild improvement of rickets.

The inhibition of FGF-23 activity with burosumab is associated with an increase in renal tubular phosphate reabsorption and the correction of hypophosphatemia in children with X-linked hypophosphatemia, which corresponds to a decrease in the severity of rickets. The healing of rickets and the improvement in the fasting serum phosphorus probably contribute to concurrent improvements in linear growth and physical activity and a reduction in pain.^13^ Burosumab likely promotes growth by treating the underlying bone disease, including improving rickets, allowing for improved development of the growth plate, and straightening of the lower limbs.^16^ Our patient’s X-rays also showed mild improvement in rickets severity (Figure 2), even though she never presented with severe rickets. Nevertheless, her height has been affected by her leg deformities, scoliosis, and her advanced bone age due to precocious puberty, which will probably result in a less favorable final height.

This is the fourth case reported in the international literature that has been successfully treated with burosumab. In 2021, Gladding et al. reported a boy with MAS treated with oral phosphorus and calcitriol since he was 14 mo old, complicated by gastrointestinal intolerance, malabsorption, hypercalciuria, and nephrocalcinosis, who commenced on burosumab at the age of 7 yr 2 mo at a dose of 1.1 mg/kg given 2 wk apart for 17 mo with great results and no adverse events.^8^ In 2022, Apperley and Senniappan reported a 13-yr-old boy with MAS with deranged biochemistry, high ALP and PTH levels from the age of 10 yr old, and ongoing leg pains despite optimal conventional therapy with oral phosphorus who did well with burosumab at a dose of 0.5 mg/kg every 2 wk for 5 wk with no short-term complications.^9^ In 2024, Sawamura et al. reported one more female pediatric patient with MAS successfully treated with burosumab for persistent hypophosphatemia due to multiple bone lesions of fibrous dysplasia.^10^ In our case, the extensive FD burden and the longstanding hypophosphatemia that was insufficiently controlled with conventional treatment made initiating burosumab treatment a necessity. Dose/interval titration was a challenge, but she is now stable with no adverse effects.

There was not a significant change in height SDS. However, given the fact that our patient is severely affected on both her legs that have also undergone surgery, we wouldn’t expect to see significant changes after 11 mo of treatment.

In conclusion, burosumab is a monoclonal antibody against FGF23, which is able to suppress FGF23 expression and reduce the risk of developing FGF23-mediated hypophosphatemia and its associated complications. Burosumab should be considered as an effective and safe alternative strategy for FGF23-mediated hypophosphatemia in FD/MAS for those who either cannot tolerate or do not respond to the association of vitamin D analog and phosphate supplements. The efficacy of burosumab for treating FD/MAS patients and the target of phosphorus levels have not been extensively elucidated yet due to the rarity of the disease. To our knowledge, this is the fourth case worldwide to have been successfully treated with burosumab. More investigations are needed, ideally as part of a clinical trial, in order to determine the potential utility of burosumab for the treatment of FGF23-mediated hypophosphatemia in patients with FD.

## Data Availability

Data are available upon request.
